# Case Report: A report on the countermeasures after the rupture of the scalp venous indwelling needle catheter in 12 cases

**DOI:** 10.3389/fsurg.2022.1056664

**Published:** 2023-01-09

**Authors:** Haiyan Zhong, Mengze Wang, Yang Gui, Lei Yang, Qianhao Zhao

**Affiliations:** Department of Neurosurgery, Kunming Children’s Hospital, Kunming, China

**Keywords:** indwelling catheter, scalp vein, fracture, countermeasures, case report

## Abstract

As a substitute for a scalp needle, the intravenous indwelling needle is easy to operate and easy to use. it is not only conducive to the rescue of critically ill children, improves nursing efficiency, but also avoids pain caused by repeated venipuncture. However, cases of indwelling needle catheter breaking and remaining in scalp vessels are rarely reported. This study collected 12 cases of scalp vein indwelling needle rupture and retention in scalp vessels in our center from January 2012 to January 2022. It was found that there were 7 males and 5 females, with an average age of 19.17 ± 8.96 months. The average length of the severed end was 15.00 ± 1.54 mm. In 8 cases, the catheter was broken during the haircut, and in 2 cases, the wall structure was damaged and broken after repeated folding of the catheter. In 2 cases, the children did not cooperate during extubation, the head twisted violently and the catheter was broken.5 cases tried to extract it by manipulation and hemostatic forceps, of which 4 cases were successful, and 1 case was successfully removed after the completion of three-dimensional computerized tomography (3D-CT) imaging positioning. The remaining 7 cases were removed by operation, and the success rate of the first operation was 100% in 4 cases who chose 3D-CT. The other 3 cases chose ordinary CT plain scan positioning, the success rate of the first operation was 66.6%, and one child was successfully removed after the second operation after the failure of the operation plus 3D-CT scan positioning. All the children were in stable condition after the operation and were discharged smoothly. When the broken catheter is relatively shallow and the scalp is not completely closed, we could choose the preliminary positioning of B-ultrasound or ordinary CT, and then try to remove it by manual squeezing combined with hemostatic forceps. However B-ultrasound and ordinary CT could not meet the requirements of accurate location, 3D-CT has a very important localization value for surgery, which can improve the success rate and help successfully remove the ruptured catheter.

## Introduction

Venous catheterization in infants and young children is quite difficult, mainly because the veins of the upper and lower limbs of infants are very small, deep, and not fully developed. However, they have less subscalp fat, clear superficial veins, and reticular distribution, and their blood can be returned through collateral circulation, whereas the scalp veins of infants are visible and palpable (1). The technology and application time of intravenous indwelling needles in children's scalps for intravenous infusion is very mature (2), commonly choosing the median frontal vein, superficial temporal vein, and retro auricular vein. The median frontal vein is shallow and thick, easy to puncture, but easy to leak during infusion. It is mainly used for small drug irritation and short-time infusion. The superficial temporal vein is shallow and clear, which is not easy to permeate and leak during infusion. The position of the retro auricular vein is deep, which is suitable for a large amount of infusion and injection of stimulant drugs. The placement time is usually 72 h–96 h, for infants and older children who need multiple transfusions, scalp intravenous infusion is indeed the first choice (3). If the indwelling needle breaks, it becomes surrounded and softened by small blood vessels and can move back and forth in the vein and embolize the vein (4). Failure of the timely removal of the retained indwelling needle will cause pain to the children and increase the psychological pressure on the medical staff and the children's families and intensify the contradiction between doctors and patients (5). In recent years, we encountered 12 cases of pediatric venous indwelling needle catheter rupture in the Department of Neurosurgery of Kunming Children's Hospital. To relate our experience and the lessons learned, we have summarized the emergency treatment and preventive countermeasures taken in the 12 cases of pediatric venous indwelling needle catheter rupture.

## Clinical information

**Figure 1:**
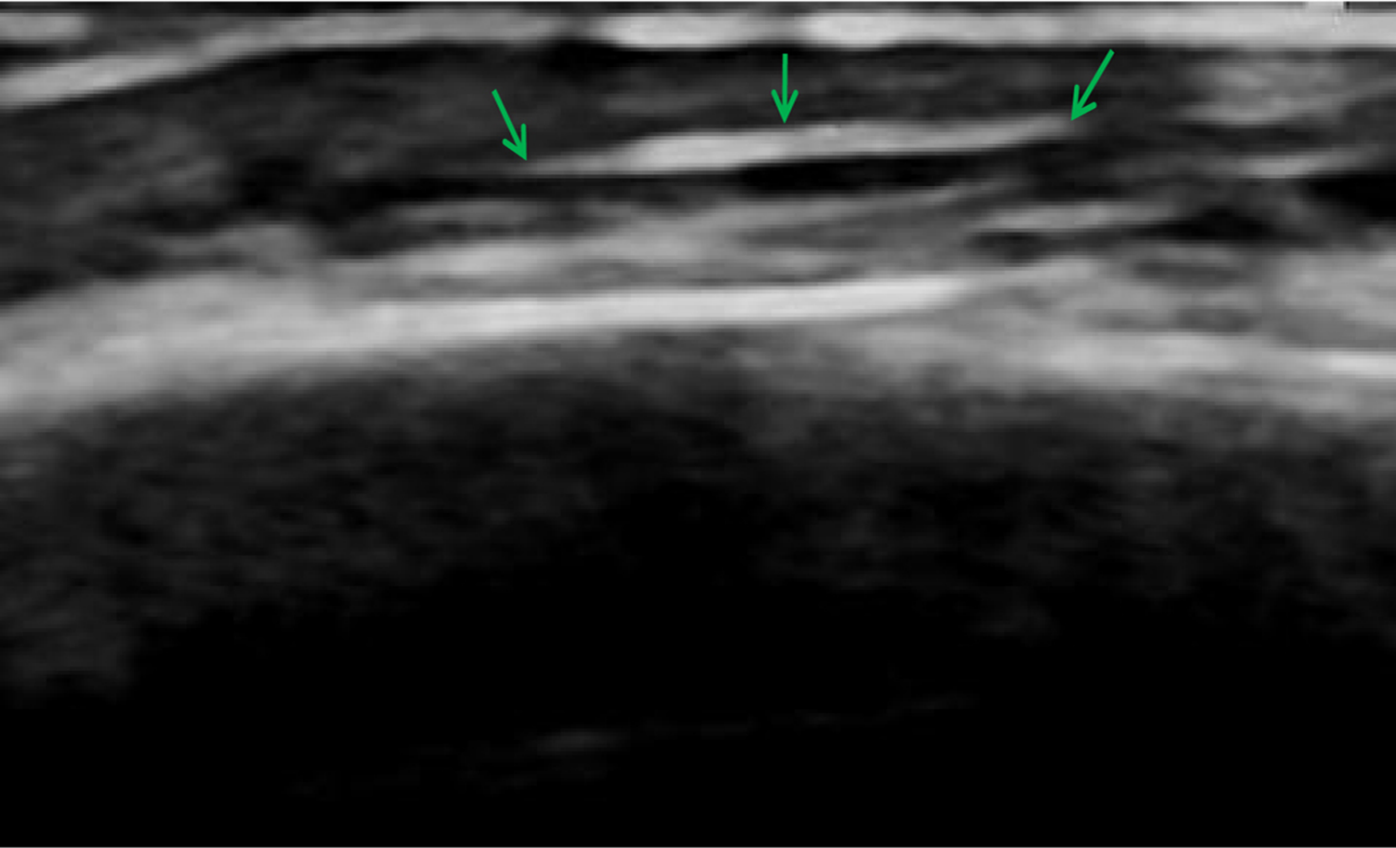
B-ultrasound confirmed that the subcutaneous indwelling needle showed a thin strip of echo under the scalp.

We collected information on 12 patients with ruptured scalp venous indwelling catheters from the Department of Neurosurgery of Kunming Children's Hospital, China from January 2012 to January 2022. The disposable intravenous indwelling needle selected in the study was provided by Suzhou Linhwa Medical Devices Co., Ltd. (Suzhou, Jiangsu Province, China). There are two kinds of specifications, the model is 24G, 26G, the length of the catheter is 16, 19 mm, the diameter of the catheter is 0.6, 0.7 mm, and the flow rate is 10, 19 ml/min. After the catheter broke, a B-ultrasound examination around the catheter indicated a thin strip of echo (Figure 1). To prevent the catheter from drifting with the blood, the vein was pressed with the middle finger of the left hand and squeezed to the distal end. Simultaneously, the skin at the puncture point was pulled to the near end of the heart with the middle finger of the right hand. After the broken catheter was fully exposed at the puncture point, the pulled skin was fixed with the index finger of the left hand, and the ruptured catheter was quickly extracted with the hemostatic forceps held in the right hand. This manual squeezing method combined with hemostatic forceps usage was applied in four cases. Manual squeezing combined with hemostatic forceps is not always effective, In some cases, surgical removal is required. Surgical precautions and procedures include the following: incision criteria include a 1 cm surgical incision in the basic location of the midpoint, not too deep, and best between the epidermis and dermis. Using small curved forceps and ophthalmic forceps, the vein is slowly separated without excessive traction; Using the belly of the finger to touch the hard cord, continue to separate and remove the broken catheter completely. making the comparison at the same time to determine whether there are any residuals. Suture 3 stitches with 5-0 silk thread and apply the bandage. The process requires patience, meticulousness, and avoiding excessive traction to avoid injury to the facial nerve.

**Figure 2:**
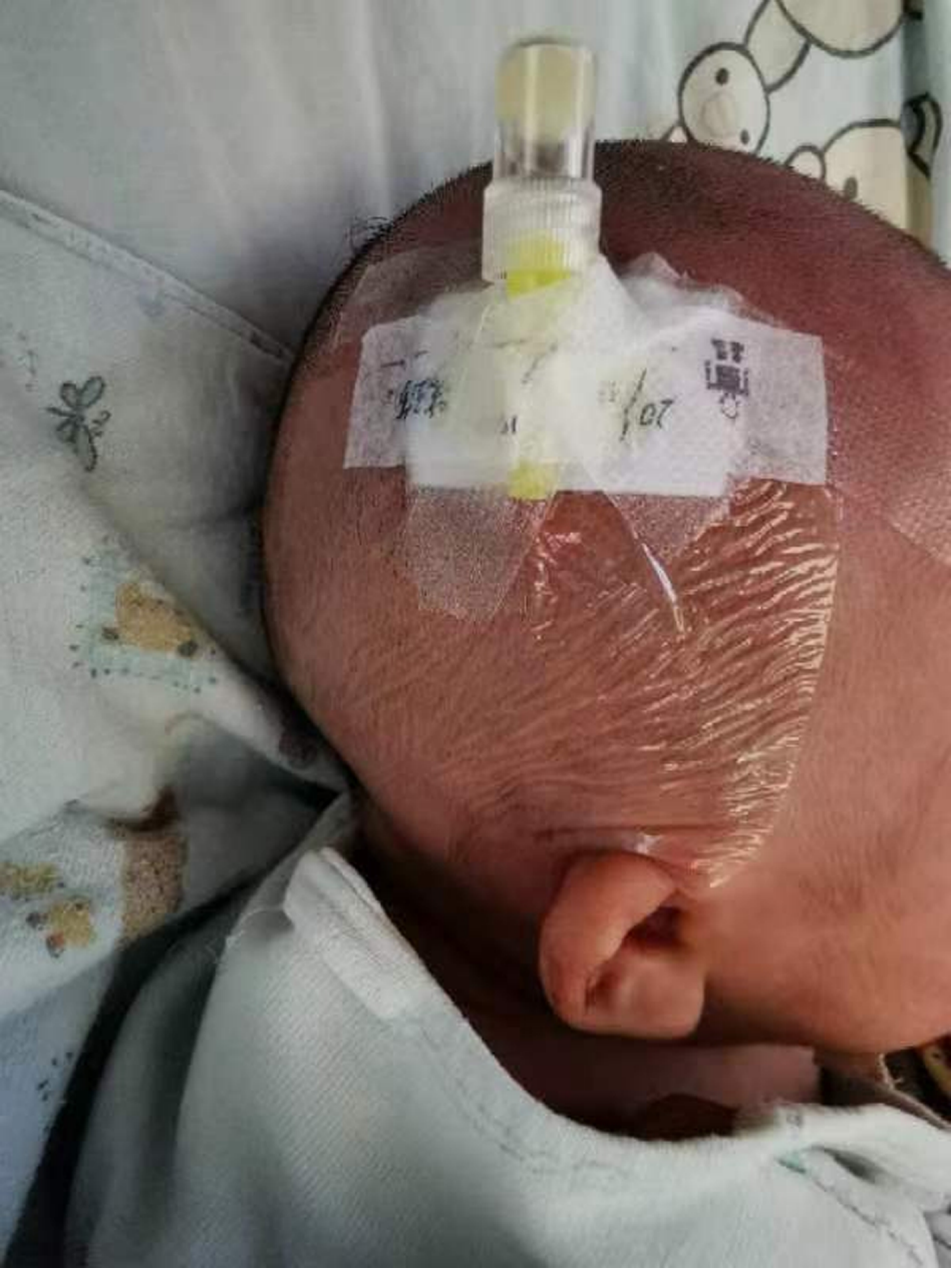
Scalp indwelling needle puncture and fixation in the right superficial temporal vein.

We reviewed the demographic data (age, sex, time of admission, etc.), location of scalp indwelling needle, cause of broken catheter, length of the broken catheter, auxiliary examination, treatment plan, and prognosis (Table 1). Of the 12 infant patients, 7 were male and 5 were female with an average age of 19.17 ± 8.96 months and the average length of the severed end at 15.00 ± 1.54 mm. there were six, four, and two cases involving the median prefrontal vein, right superficial prefrontal vein, and right superficial temporal vein (Figure 2), respectively. In eight cases, as no effective safety measures were taken during head shaving, the indwelling needle catheter was broken at the puncture point when the patient's head turned suddenly and the hairdresser inadvertently touched the indwelling needle. In two cases, the fluid dripping was not smooth during infusion, although there was no redness and swelling at the puncture site. The indwelling needle catheter became twisted and folded causing a dripping obstruction. After this folding was released, the indwelling needle continued unobstructed infusion. The dripping obstruction occurred many times, and the infusion patency was restored by pressing or pulling the indwelling needle stem, which caused the catheter to break. In the remaining two cases, the infants became agitated during extubation and the catheter broke when they twisted their heads violently during the process.

**Table 1:** Information of 12 patients with rupture of scalp venous indwelling needle catheter.

Patient number	Sex/Age (month)	Admission time	Position	Reasons of pipe break	Supplementary Examination	Length(mm)	Treatment
1	Male/15	2012	Right forehead superficial vein	Improper use of razor	Bedside ultrasound	15	Squeeze out with hemostatic forceps
2	Male/8	2017	Median frontal vein	Improper use of razor	Bedside ultrasound	17	Squeeze out with hemostatic forceps
3	Male/35	2019	Right superficial temporal vein	Do not cooperate with extubation	Bedside ultrasound	15	Squeeze out with hemostatic forceps
4	Female/9	2015	Median frontal vein	Improper use of razor	Bedside ultrasound	15	Squeeze out with hemostatic forceps
5	Male/29	2019	Right forehead superficial vein	Improper use of razor	Bedside ultrasound + 3D-CT	16	First surgery to take out
6	Female/21	2013	Median frontal vein	Do not cooperate with extubation	ordinary CT	14	First surgery to take out
7	Male/17	2014	Median frontal vein	Catheter folding	ordinary CT	12	First surgery to take out
8	Female/18	2014	Right forehead superficial vein	Improper use of razor	3D-CT	17	First surgery to take out
9	Male/12	2015	Median frontal vein	Improper use of razor	3D-CT	13	First surgery to take out
10	Male/13	2016	Median frontal vein	Improper use of razor	ordinary CT + 3D-CT	16	Second surgery to take out
11	Female/33	2018	Right superficial temporal vein	Catheter folding	3D-CT	14	First surgery to take out
12	Female/20	2021	Right forehead superficial vein	Improper use of razor	3D-CT	16	First surgery to take out

CT, Computed Tomography.

**Figure 3:**
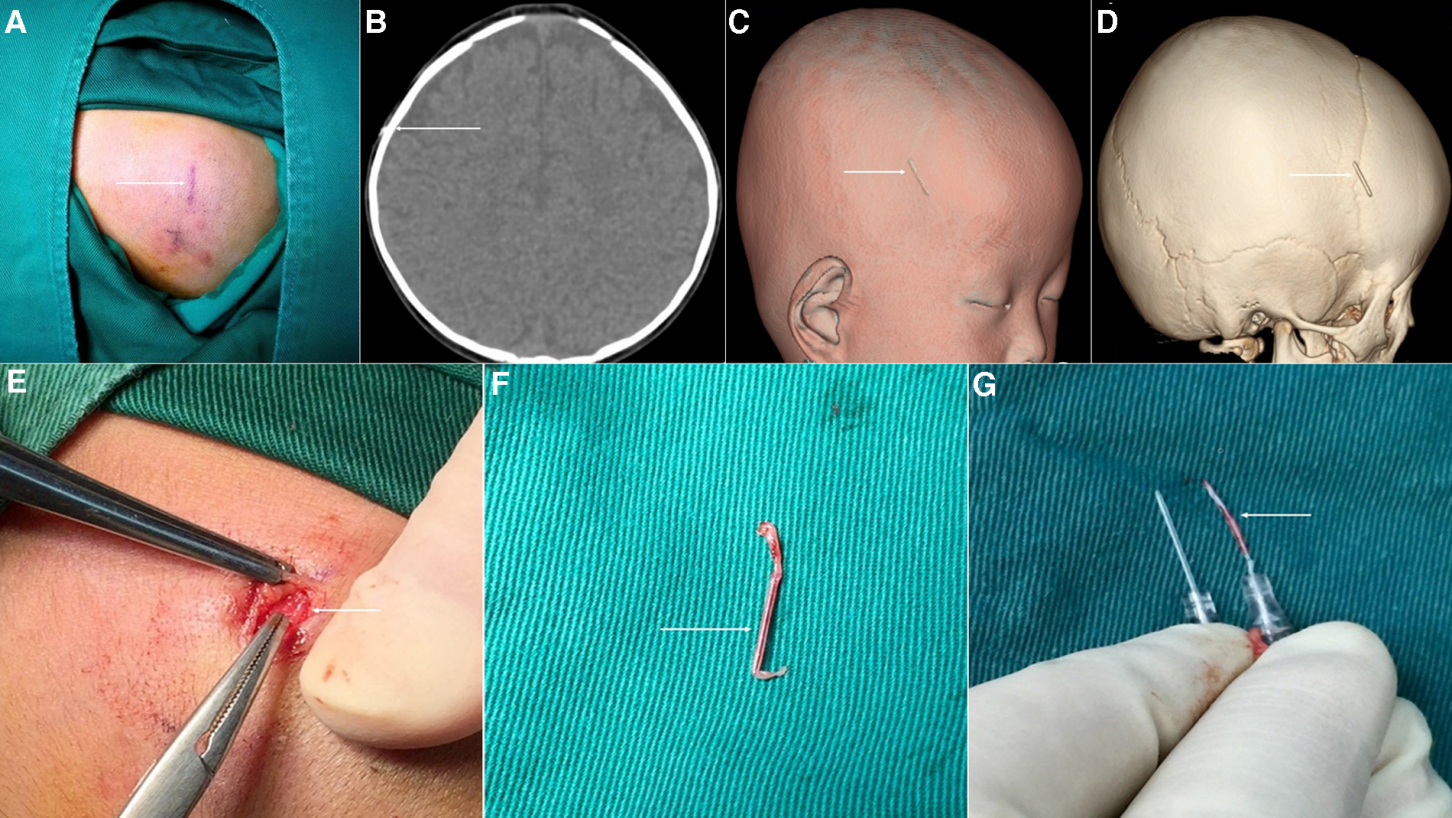
Surgical removal of the indwelling needle from the broken scalp vein. (**A**) The local puncture area of the skin near the superficial forehead vein is red and swollen with cord-like changes. (**B**) The broken vein indwelling needle is seen as high density on ordinary CT scan. (**C,D**) 3D-CT imaging clearly shows the specific location of the fracture, as well as the surrounding bone markers. (**E**) Blood vessels are seen completely wrapped around the broken indwelling needle. (**F**) The broken catheter was removed successfully during the operation. (**G**) The broken end coincides with the residual needle stem, which is consistent with the length of the normal indwelling needle.

In 5 cases, Manual squeezing and hemostatic forceps were attempted, but only 4 cases were successful. The remaining case was successfully removed by the first operation after 3D-CT imaging localization. This case involved a 2-year-old male infant who was treated with a scalp vein indwelling needle after hospitalization. The puncture site was in the superficial forehead vein on the right forehead, which was completely covered with the medical film and the indwelling needle was attached. During the removal of the indwelling catheter, it was difficult to tear it off because of the tight adhesion between the medical film and the hair around the indwelling needle site. To avoid pain caused by forcibly tearing off the film, the nurse first used an infant electric hair clipper to shave the surrounding hair and pulled out the indwelling needle catheter. It is suspected that the catheter may have become damaged during shaving, resulting in a fracture during extubation, leaving the stump in the superficial vein of the scalp. After the bedside B-ultrasound scan confirmed the same, manual squeezing was attempted but it failed. The local skin redness and cord-like changes could be seen for approximately 5 days after the indwelling needle broke (Figure 3A). The high-density shadow of the vein indwelling needle under the right forehead scalp could be observed on ordinary CT, although a precise localization was difficult (Figure 3B). 3D-CT imaging clearly showed the location of the blood vessels and related bony markers in the scalp blood vessels (Figures 3C,D). Using 3D-CT imaging for guidance, the epidermis was cut during the operation; the vein was filled, red, and slightly hard when it was slowly separated, and blood vessels were observed to be wrapped around the broken indwelling needle (Figure 3E). The length was 16 mm, which was consistent with that of the residual needle stem and the length of the normal indwelling needle (Figures 3F,G).

In the other seven children, considering the difficulty of Manual squeezing combined with the usage of hemostatic forceps to remove the broken end, the broken indwelling catheter was removed under general anesthesia. Among them, four children underwent 3D-CT scanning, and the ruptured catheter was removed successfully for the first time operation. The other three patients were scanned by ordinary CT; among these, the first operation succeeded in removing the broken catheter in two cases but failed in one case, wherein the broken catheter was relocated by 3D-CT imaging and removed by a second operation. So, the success rate of the first operation using 3D-CT was 100% in four of the remaining seven cases, and that using ordinary CT was 66.6% in three cases.

## Discussion

The intravenous indwelling needle is made of soft polytetrafluoroethylene or biomaterials that are characterized by softness and toughness (6). This needle is widely used for clinical infusion in children in the outpatient and emergency departments (7). The median frontal vein, superficial temporal vein, retro auricular vein are usually selected for scalp vein indwelling needle puncture (8). The improper use of a shaver can lead to the rupture of residual scalp vessels with a venous indwelling needle (9). We found that the common causes of catheter rupture were that the hair was not completely shaved before the scalp vein was punctured, the electric razor was used improperly, or the children were agitated during extubation (10). Timely removal of the indwelling needle catheter was difficult when it became folded or twisted. While extracting the indwelling needle, if the patients moved their heads violently, the indwelling needle catheter folded repeatedly, damaging the wall structure of the indwelling needle, and thus, an inability to locate the catheter fracture the first time. This caused the loss of valuable time to extract the broken catheter, resulting in fracture (11). Therefore, when choosing scalp venipuncture, the hair covered by the dressing should be fully shaved to ensure that the dressing is fully suited to the skin. The dressing should not be pasted on the child's hair as it increases the difficulty of needle extraction. Moreover, excessive winding tape to cover the puncture site should also be avoided during fixation. On the outside of the indwelling needle, an elastic mesh bandage cap or self-adhesive bandage is used for auxiliary fixation to increase the fixation effect and prevent the indwelling needle from slipping (12). The puncture site should be evaluated every day, and if the dripping during infusion is suspected to be obstructed, the cause should be carefully checked. Adjusting the angle of the needle stem to straighten out the folding part or pulling the needle stem should not be done. even fold the catheter and then send it into the vein to continue to maintain the infusion.

Among the cases described in this report, there were eight cases of catheter fracture caused by the improper use of the shaver, accounting for 66.7%. As the indwelling needle hose itself is a foreign body, after its fracture, it is trapped in the scalp vein and the surrounding blood vessels and tissue become completely wrapped around the needle, resulting in venous thrombosis and phlebitis. When the venous indwelling needle remains broken inside for more than 5 days, phlebitis occurs locally, the skin around the indwelling needle hose becomes slightly red, and a cord-like stiff feeling can be felt. When the broken end is shorter or flows deeper with the blood, the injury increases, the foreign body sensation may not be obvious, and it is more difficult to remove. The failure to extract it before or after the first operation will cause a great burden to the family members of the patients and medical staff of the relevant hospitals. For a broken catheter, bedside B-ultrasound is a quick and simple examination method (13). In the current study, after the initial localization by B-ultrasound, emergency manual squeezing and hemostatic forceps were successfully used to remove the broken catheter in four cases before the puncture point was closed. In the fifth case, the puncture point was closed, manual removal failed, and an operation succeeded in catheter removal using 3D-CT imaging. Therefore, after a catheter fracture is first discovered, the broken catheter needs to be removed as soon as possible before the puncture point is closed.

Of the three examination methods, the B-ultrasound is the fastest and most convenient. Ordinary CT can detect high-density foreign bodies under the scalp, although precise localization of foreign bodies is difficult. 3D-CT imaging can accurately locate the ruptured catheter and judge the position of the catheter from many levels, dimensions, and bony markers, which is of great significance for the success of the operation (14). In the early stage, we also chose B-ultrasound or ordinary CT to help locate the residual broken catheter. When we conduct a physical examination, the broken end is relatively shallow and the scalp is not completely closed, we could choose the preliminary positioning of B-ultrasound, and then try to remove it by manual squeezing. But sometimes the broken catheter is very small, the incision is only about 1 cm, and B-ultrasound or ordinary CT can not be located accurately, resulting in difficult surgical removal. The infant's cranial suture is easy to touch. According to the results of 3D-CT, we judge the relationship and distance between the indwelling needle and the upper edge of the auricle and the cranial suture, to determine the position of the broken indwelling needle under the scalp, and mark it on the surface of the scalp, which is the location of the surgical incision. So, 3D-CT can help us make the operation more smooth. In our case, 3D-CT imaging-assisted surgery achieved a success rate of 100%. The operation is generally carried out under anesthesia after the primary disease of the child is controlled.

Finally, the choice of a high-quality indwelling needle catheter is crucial, ensuring high-quality material with good spring back performance and a material that is not easily breakable (6, 15). In the event of a broken venous indwelling needle, it is important to handle the situation calmly, fully communicate with the family members, appease the mood, and remove the puncture point by pushing from the distal end and using hemostatic forceps as soon as possible before the puncture wound closes. If this manual squeezing is unsuccessful, the indwelling needle can be removed under general anesthesia after the primary disease has improved. 3D-CT scanning technology can further determine the location of the vein where the foreign body is located and the surrounding bone marks by adjusting the parameters; it has obvious advantages compared with B-ultrasound and ordinary CT plain scan and can be used as the first choice of auxiliary examination. Therefore, 3D-CT should be given priority once surgical management is decided, which can improve the success rate of surgery and help successfully remove the ruptured catheter.

## Data Availability

The original contributions presented in the study are included in the article/Supplementary Material, further inquiries can be directed to the corresponding author/s.
